# Antiaging Activity of Peptide Identified from Fermented *Trapa Japonica* Fruit Extract in Human Dermal Fibroblasts

**DOI:** 10.1155/2020/5895029

**Published:** 2020-04-29

**Authors:** Jin Dong Jang, Minkyung Kim, Gun-He Nam, Young-Min Kim, Sang Moon Kang, Kee-Young Lee, Young-Joon Park

**Affiliations:** ^1^Doori Cosmetics Co., Ltd., 13F Galaxy Tower, 175 Saimdang-ro, Seocho-gu, Seoul, Republic of Korea; ^2^Department of Biological Science and Biotechnology, College of Life Science and Nano Technology, Hannam University, 1646 Yuseong-daero, Yuseong-gu, Daejeon 34054, Republic of Korea; ^3^A&PEP INC., 13, Oksansandan1-ro, Oksan-myeon, Heungdeok-gu, Cheongju-si, Chungcheongbuk-do, Republic of Korea; ^4^College of Pharmacy, Ajou University, 206, World Cup-ro, Yeongtong-gu, Suwon-si, Gyeonggi-do, Republic of Korea

## Abstract

We have previously shown that *Trapa japonica* fruit extract (TJE) as well as its fermented extract (FTJ) can be potentially used to treat alopecia. In the current study, a newly synthesized peptide (PEP) was detected in an active compound isolated from FTJ. Several biological assays were conducted to verify the antiaging effects of TJE, FTJ, and PEP on the skin. We examined the effects of TJE, FTJ, and PEP on cell viability, collagen synthesis, and inhibition of mRNA expression of matrix metalloproteinases (MMPs), induced by tumor necrosis factor alpha (TNF-*α*), in human dermal fibroblasts (HDFs). In addition, a wound-healing assay of the human keratinocyte cell line (HaCaT) and a clinical study of antiaging activity were conducted. The findings confirmed that PEP exerted an effect on cell proliferation in a dose-dependent manner. Treatment with TJE, FTJ, and PEP increased collagen synthesis but inhibited TNF-*α*-induced mRNA expression of MMPs. Compared with TJE and FTJ, PEP promoted a significant level of wound recovery in HaCaT cells and also exhibited antiaging effect, as demonstrated by a clinical study. These results suggest that PEP shows potential as a skin antiaging cosmetic product.

## 1. Introduction

Skin aging is a complex biological process involving both intrinsic and extrinsic factors [1]. Intrinsic aging is due to inevitable, natural processes associated with genetics, hormones, and cellular metabolism, resulting in increased fine wrinkles and skin surface roughness as well as thin and dry skin. Extrinsic aging, which is characterized by coarse wrinkles, furrows, roughness, dryness, sagging skin, laxity, loss of elasticity, and pigmentation, is induced by long-term accumulation of external environmental factors such as sun exposure, pollution, smoking, and improper lifestyle [2, 3].

Several studies have reported that skin aging occurs via a variety of molecular mechanisms including oxidative stress, DNA damage and mutations, telomere shortening, inflammation, and the accumulation of advanced glycation end products (AGEs) [4–6]. Furthermore, reactive oxygen species (ROS) cause skin aging by stimulating the expression of matrix metalloproteinases (MMPs) that contribute to skin aging by degrading various components of extracellular matrix (ECM) proteins such as collagen, fibronectin, elastin, and proteoglycans [7, 8].

Interest in antiaging products is rapidly increasing due to a significant growth in the aging population. As a result, focus on antiaging skin products has increased, with particular reference to the thriving cosmetics market [9]. Thus, studies leading to the development of functional antiaging cosmetic products using natural ingredients, that are neither toxicants nor skin irritants, are actively being pursued [10].

Peptides are considered as useful cosmetics material, because they are light and air stable, less toxic, exhibit a strong affinity for water, and possess moisturizing capacity [11, 12]. They are widely used as ingredients in functional cosmetic products with antioxidant, anti-inflammation, collagen synthesis, antiwrinkle, whitening, and wound-healing effects which are intended to improve skin conditions [13–15]. Recently, the development of novel natural peptides, as well as more stable and effective synthetic peptides, has raised interest in the use of peptide-based cosmetics in skin care [16, 17].

*Trapa japonica*, a well-known aquatic plant, belongs to the genus *Trapaceae*. It is rooted in the water, while its thin, long stems containing aerocysts float on water. It is mostly found in shallow water fields, reservoirs, or ponds around the country [18]. It contains high starch levels, many dietary fibers, and polyphenols, such as trapain, ellagic acid, eugeniin, and gallic acid [19, 20]. Previous studies have shown that *T. japonica* exhibits antidiabetic activity in addition to exerting antioxidant and anti-inflammatory effects. It also exerts inhibitory effects on ultraviolet B- (UVB-) induced expression of 11*β*-hydroxysteroid dehydrogenase type 1 (11*β-HSD*1) [21–24]. Previously, our studies indicated that the active ingredients of the *T. japonica* fruit extract were supplemented by fermentation with two microorganisms: *Bacillus subtilis* and *Bacillus methylotrophicus* [25, 26]. We additionally demonstrated that the fermented *T. japonica* fruit extract induced the proliferation of human hair follicle dermal papilla cells.

In this study, we examined compounds isolated from the fermented *T. japonica* fruit extract and synthesized a new peptide, which was identified structurally. Furthermore, we investigated the antiaging effect of the newly synthesized peptide via in vitro tests and a clinical study.

## 2. Materials and Methods

### 2.1. Materials and Reagents

Trifluoroacetic acid (TFA) was purchased from Sigma-Aldrich Co., (St. Louis, MO, USA. Ethanol, methanol, hexane, chloroform, ethyl acetate, n-butane, and HPLC-grade water were purchased from Duksan Chemicals Co. (Seoul, Korea)). All samples were filtered using the PTFE membranes (0.2 *μ*m; Adconcinc MFS Inc.) before injection. Human keratinocyte-derived HaCaT cells and human dermal fibroblasts (HDFs) were obtained from the American Type Culture Collection (ATCC, Manassas, VA, USA).

### 2.2. Plant Material and Fermentation

*T. japonica* fruits (Voucher number: KP255650) were obtained from China. Next, 500 g of dried *T. japonica* fruit was extracted with 5000 mL of distilled water for 24 h at 50°C and filtered using a filter funnel and filter paper. 3.67% (w/w) of *T. japonica* fruit was obtained as an extract (TJE). Fermentation of TJE was performed using *B. subtilis* (K007) and *B. methylotrophicus* (S001), which were separately identified by A&PEP Inc (Cheongju, Korea). After preculturing in nutrient broth (Difco, Detroit, MI, USA) for 24 h with shaking in an incubator at 37°C, the two microorganisms were inoculated into the medium (0.6% glucose, 0.3% yeast extract, and 0.1% soytone) containing TJE in a fermenter ( FMT ST-D-14; Fermentec, Korea) at 37°C for 3 d. The final fermentation product was filtered using a 0.2 *μ*m filter.

### 2.3. Fraction and Isolation

The fermented *T. japonica* fruit extract (FTJ) was sequentially fractionated with hexane, chloroform, ethyl acetate, butanol, and water. The water fraction was concentrated using a rotary vacuum concentrator (RE100-pro; DLAB Scientific Inc., USA). Six fractions were separated using a Sephadex-LH20 column and a RP silica column in sequence and were purified via high-performance liquid chromatography (HPLC). HPLC was performed using a Waters 2690 Separation Module and a Waters 2487 Dual λ Absorbance Detector. The analytical column (Xterra C18 ODS, 0.5 × 250 nm) was packed with LiChrospher 100RP-18 (15 *μ*m; Merck Co.). The final compound obtained via a further HPLC process was confirmed to be a tripeptide by nuclear magnetic resonance (NMR; 1H, 13C, 2D-NMR).

### 2.4. Peptide Synthesis

The peptide (PEP) was synthesized using 3 amino acids. Swelling was induced via the addition of 2-chlorotrityl chloride resin and methylene chloride (MC), following which an amino acid and diisopropylethylamine (DIPEA) were also added, together with dimethyl formamide (DMF). Next, a deblocking solution was added and the preparation was washed using MC. A second amino acid was allowed to react with the mixture of *N*-hydroxybenzotriazole (HOBt) and *O*-(benzotriazol-1-yl)-*N,N,N′,N′*-tetramethyluranium hexafluorophosphate (HBTU) dissolved in DMF and DIPEA. Next, deblocking solution was added and the preparation was washed using MC. A third amino acid was also allowed to react via the same method. The reaction mixture was concentrated and purified using HPLC.

### 2.5. WST-1 Assay

Cell proliferation was determined using water-soluble tetrazolium salt (WST-1; EZ-CytoX, Daeil Lab Service, Korea). HDFs were cultured in Dulbecco's Modified Eagle's Medium (DMEM; Welgene, Korea) containing 10% fetal bovine serum (FBS) for 24 h in a humidified incubator under 5% CO_2_ at 37°C. Cells were treated with a range of test sample concentrations (0-1 *μ*g/mL) and incubated for 24 h. Absorbance was measured using an ELISA microplate reader (Bio-Rad model 680; Bio-Rad Laboratories Inc., Japan) at 450 nm.

### 2.6. Measurement of Collagen Synthesis

HDFs were cultured in DMEM supplemented with 10% FBS for 24 h in a humidified incubator under 5% CO_2_ at 37°C. The medium was replaced with a serum-free medium and treated with 1 *µ*g/mL of TJE, FTJ, and PEP for 24 h. Collagen synthesis in cell supernatants was quantified using a collagen type 1 c-peptide ELISA kit (Takara Bio Inc.) in accordance with the manufacturer's protocol.

### 2.7. Measurement of Reverse Transcription-PCR

mRNA expression levels of MMP-1 and MMP-9 were determined via reverse transcription-polymerase chain reaction (RT-PCR). Following pretreatment with 1 *µ*g/mL of TJE, FTJ, and PEP for 1 h, HDFs were treated with 10 ng/mL of TNF-*α* for 24 h. Isolation of total RNA from HDFs was performed using RiboEx™ solution (GeneAll Biotechnology, Korea). One microgram of RNA was reverse transcribed to complementary DNA (cDNA) using a DiaStar™ RT kit (SolGent, Seoul, Korea). RT-PCR was performed via Rotorgene 3000 real-time PCR (Corbett Research, UK) using PowerUp SYBR™ Green Master Mix (Takara, Korea) and primers in accordance with the manufacturer's instructions. Glyceraldehyde-3-phosphate dehydrogenase (*GAPDH*) was used as the internal control for data normalization. The following primers were used: *MMP-1*, 5′-TTTTAATGGGCAGGAGATGC-3′ and 5′-GGATGATGAAAAGGCTGGAA-3′; *MMP-9*, 5′-GAGACCGGTGAGCTGGATAG-3′ and 5′-TACACGCGAGTGAAGGTGAG-3′; *GAPDH*, 5′-TTCCTCGGTGATACCCACTC-3′ and 5′-AGGACCTTCCCGTTTCACTT-3′.

### 2.8. Wound-Healing Assay

HaCaT cells were grown in DMEM supplemented with 10% FBS at 37°C in an incubator under 5% humidified CO_2_ for 24 h. The monolayer of cells was scratched with a 10 *μ*L sterile pipette tip, following which the cells were washed with phosphate buffered saline (PBS) to remove detached cells. Next, cells were treated with the test samples (1 *μ*g/mL) for 24 h. The area of wound repair was assessed under a light microscope (Carl Zeiss, Thornwood, NY, USA) equipped with a digital camera.

### 2.9. Clinical Trial to Determine the Antiaging Effect

The clinical study was approved by the local ethics committee (KDRI-IRB-18659, Korea) and performed in compliance with the Declaration of Helsinki and Good Clinical Practice guidelines (GCP). All procedures were performed in accordance with ethical standards. Informed consent was obtained from all participants (22 healthy women, aged 41 to 57 years). The subjects applied the test sample (eye cream containing 0.5% PEP) to their face twice a day for 8 weeks. The components of the eye cream are shown in Table 1. The experimental design comprised a randomized, double blind test. All subjects washed their faces using a standard cleanser and rested under optimal conditions (temperature 20–24°C, relative humidity 40–60%) for 30 min before being subjected to skin analyses. The skin condition of subjects was evaluated by dermatologists. The antiaging effect of the test sample was evaluated using 5 parameters (R1, skin roughness; R2, maximum roughness; R3, average roughness; R4, smoothness depth; R5, arithmetic average roughness) via a Skin-Visiometer SV600 (CK electronic GmbH, Germany). Clinical data were statistically analyzed using Minitab 18 (Minitab 18.1®; Minitab Inc.) program.

### 2.10. Statistical Analysis

All data were expressed as mean ± standard deviation of three independent experimental replicates. Statistical significance was determined by Student's *t*-test. Statistical significance was set at either *p* < 0.05 or *p* < 0.01 symbolized by *∗* and *∗∗*, respectively.

## 3. Results and Discussion

### 3.1. Result

#### 3.1.1. New Peptide Synthesis Identified from FTJ

**Figure 1:**
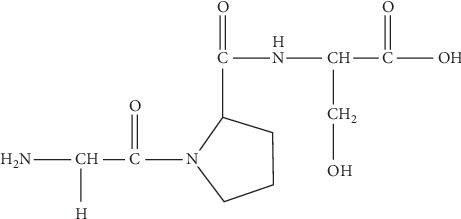
The structure of a peptide derived from fermented *Trapa japonica* fruit extracts.

As previously stated, fermentation of TJE using *B. subtilis* (K007) and *B. methylotrophicus* (S001) yielded FTJ. In our recent study, we isolated a tripeptide from FTJ [26]. In brief, the fermented biomass of *T. japonica* was extracted by using water as a solvent, and the extract was fractionated using silica gel and Sephadex LH-20 columns. The fraction with the highest protein content was selected, and further separation of the fraction was conducted through the RP silica column. Using HPLC analysis, the peptide AC2, which was a pure single peptide, was finally isolated [26]. An in vitro efficacy test indicated that this tripeptide exhibited skin-related bioactivities. A novel peptide (PEP) was synthesized from this tripeptide and subsequently identified (Figure 1). This novel PEP was synthesized using three amino acids, and its purity was confirmed by HPLC analysis to be 96.87%.

#### 3.1.2. Effects of TJE, FTJ, and PEP on Cell Proliferation and Collagen Production

**Figure 2:**
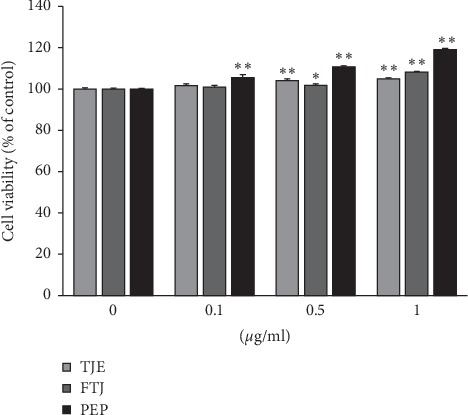
Effects of TJE, FTJ, and PEP on cell viability in HDFs. Cells were treated with various doses of TJE, FTJ, and PEP (0, 0.1, and 0.5, 1 *μ*g/mL) for 24 h. The proliferative effects of TJE, FTJ and PEP were evaluated via a WST-1 assay. Data are presented as mean ± standard deviation of three independent experiments. ^*∗*^*p* value < 0.05; ^*∗∗*^*p* value<0.01 vs. untreated group.

**Figure 3:**
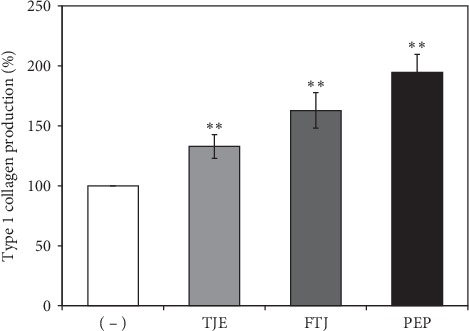
Effects of TJE, FTJ and PEP on collagen production in HDFs. Cells were cultured with TJE, FTJ, and PEP at a concentration of 1 *μ*g/mL for 24 h. The supernatant was collected from each well and procollagen type 1 c-peptide (PIP) was measured using an ELISA kit. Data are presented as mean ± standard deviation of three independent experiments ^*∗*^*p* value < 0.05; ^*∗∗*^*p* value<0.01 vs. control group.

We evaluated the effects of TJE, FTJ, and PEP on human dermal fibroblast (HDF) proliferation. The cells were treated with various concentrations (0-1 *μ*g/mL) of these 3 substances, following which cell viability was determined via the WST-1 assay. No cytotoxicity was observed up to 1 *μ*g/mL of either TJE, FTJ, or PEP (Figure 2). Furthermore, only PEP showed significant cell proliferation activity in a dose-dependent manner. In order to investigate the effects of TJE, FTJ, and PEP on collagen synthesis, HDFs were pretreated with 1 *μ*g/mL of TJE, FTJ, and PEP for 24 h and the supernatant was analyzed using a collagen type 1 c-peptide (PIP) ELISA kit. The results indicated that TJE and FTJ increased collagen production by 33% and 63%, respectively, compared to the control group (Figure 3). PEP increased collagen synthesis by 95% which was a significantly higher increase compared to that observed in the control group. Therefore, these results demonstrated that TJE, FTJ, or PEP may protect against skin aging by enhancing collagen production.

#### 3.1.3. Inhibitory Effects of TJE, FTJ, and PEP on MMP-1 and MMP-9 Expression Induced by TNF-*α*

**Figure 4:**
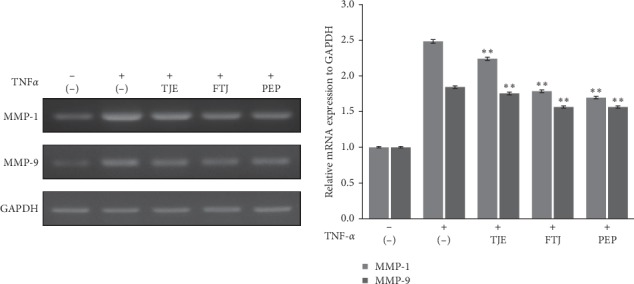
Effects of TJE, FTJ, and PEP on TNF-*α*-induced MMP-1 and MMP-9 mRNA levels in HDFs. Cells were pretreated with TJE, FTJ, and PEP at a concentration of 1 *μ*g/mL for 1 h and then stimulated with 20 ng/mL TNF-*α* for 24 h. The mRNA expression levels of MMP-1 and MMP-9 were measured using RT-PCR. GAPDH was used as the internal control. ^*∗*^*p* value < 0.05; ^*∗∗*^*p* value<0.01 vs. TNF-*α*-treated group.

The inhibitory effects of TJE, FTJ, and PEP on the mRNA expression of MMPs, induced by TNF-*α* in HDFs are shown (Figure 4). Cells were pretreated with 1 *μ*g/mL of TJE, FTJ, and PEP and then treated 20 ng/mL TNF-*α* for 24 h. The mRNA expression of *MMP-1 and MMP-9* was determined via RT-PCR. The findings confirmed that TNF-*α* induced a significant increase in the expression of MMP-1 and MMP-9 mRNA compared with that of the control group (untreated). TJE and FTJ decreased the mRNA expression levels of TNF-*α*-induced MMP-1 and MMP-9. However, PEP induced a particularly significant reduction in the mRNA expression levels of MMP-1 and MMP-9 increased by TNF-*α*.

#### 3.1.4. Effects of TJE, FTJ, and PEP on Wound Recovery

**Figure 5:**
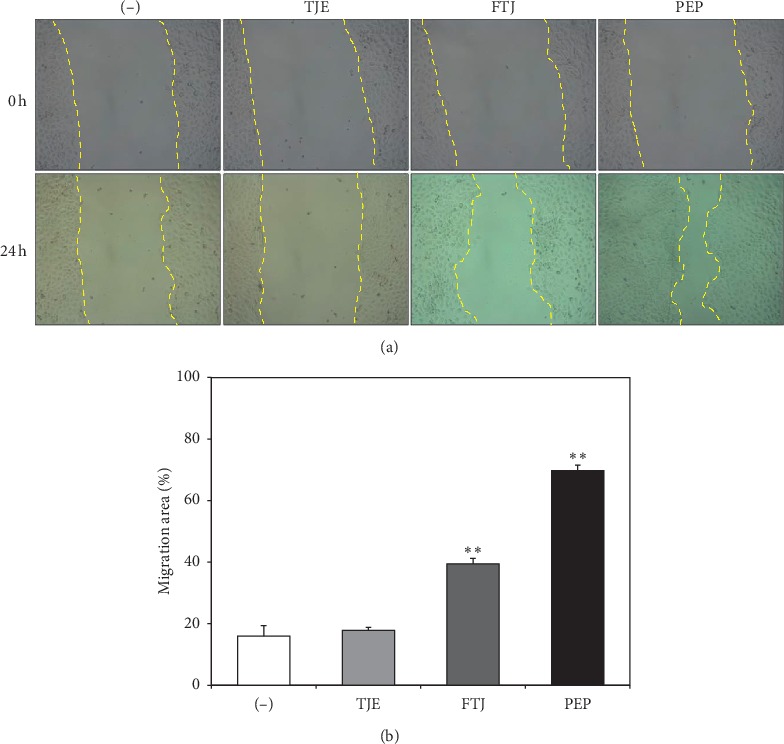
In vitro wound-healing assay of HaCaT. A wound-healing assay was performed to observe the effects of TJE, FTJ, and PEP treatment on HaCaT wound recovery. Cell monolayers were scratched and treated with TJE, FTJ, and PEP (1 *μ*g/mL) for 24 h. Cell migration into the wound was examined via light microscopy. (a) Yellow dotted lines represent the wound boundary. Quantitative analysis of the migration area was obtained via a wound-healing assay of HaCaT. (b) Data are presented as mean ± standard deviation of three independent experiments. ^*∗*^*p* value < 0.05; ^*∗∗*^*p* value<0.01 vs. control group.

Wound recovery activities of TJE, FTJ, FTE and PEP were evaluated in a human-derived keratinocyte cell line (HaCaT) via a scratch wound-healing assay. HaCaT cells scratched by using the pipette tip were treated with 1 *μ*g/mL of TJE, FTJ, and PEP for 24 h. Both FTJ and PEP exhibited wound-recovery activities, whereas TJE did not (Figure 5). Recovery of the scratched area in PEP-treated cells, in particular, was enhanced by 4.4-fold, which was significant compared to that in the control group.

#### 3.1.5. Antiaging Effect of PEP Observed in Clinical Study

**Figure 6:**
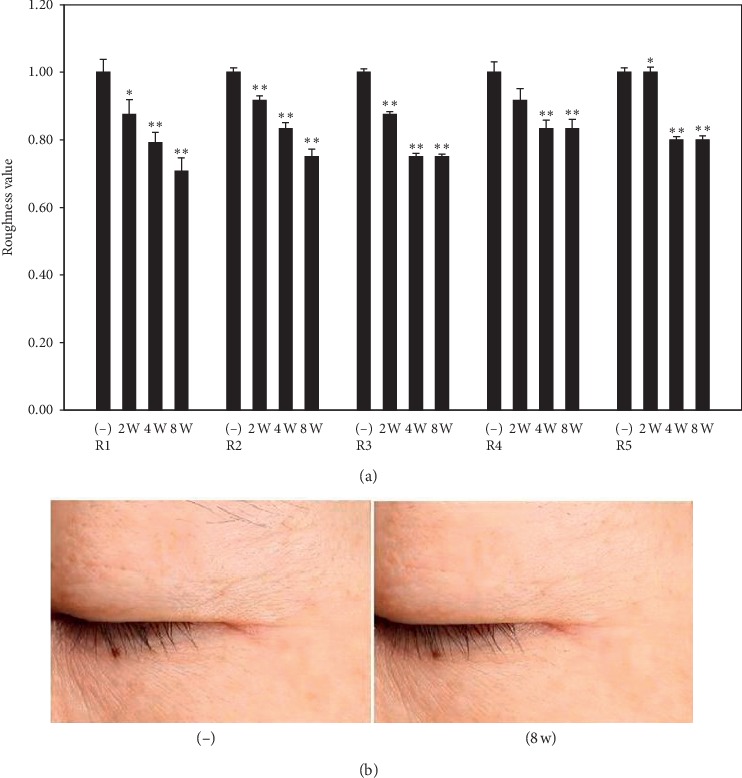
Clinical study on the antiaging effect of PEP. A formulation containing 0.5% PEP was applied to the face of 22 female subjects twice a day for 8 weeks. (a) Roughness parameters (R1, skin roughness; R2, maximum roughness; R3, average roughness; R4, smoothness depth; R5, arithmetic average roughness) and (b) representative photographs. ^*∗*^*p* value < 0.05; ^*∗∗*^*p* value<0.01 vs. control group.

Antiaging activity of PEP was evaluated at the clinical trial level using an eye cream containing 0.5% PEP. Twenty-two older female volunteers with skin wrinkles were selected from the group. A randomized double-blind test was performed, wherein a test, the sample was applied to the facial area twice a day for 8 weeks. Antiaging effect of PEP was evaluated using a skin visiometer which measured wrinkle parameters as follows: R1, skin roughness; R2, maximum roughness; R3, average roughness; R4, smoothness depth; and R5, arithmetic average roughness. Results demonstrated that all wrinkle parameters associated with the facial area were significantly decreased when the eye cream containing 0.5% PEP was used for 8 weeks (Figure 6(a)). Additionally, image analysis confirmed that skin wrinkles were reduced by application of the test sample for 8 weeks (Figure 6(b)). Thus, the current study indicated that PEP exerted an inhibitory effect on skin wrinkling.

### 3.2. Discussion

The current study investigated the effect of PEP on antiaging via in vitro clinical studies. In a previous study, we established a water-fermentation process using two microorganisms, *B. methylotrophicus* and *B. subtilis*, and conducted solvent fractionation using hexane, chloroform, ethyl acetate, butanol, and water to separate biologically active materials from FTJ [25]. Among the bioactive substances obtained from FTJ, peptides were selectively explored because peptides are extremely effective as ingredients of cosmetic skin-care products due to biological effects such as pigmentation, anti-inflammation, cell proliferation, wound healing, angiogenesis, and antiaging [13]. In addition, peptides are attracting attention as cosmeceutical materials that are suitable for use in antiaging skin care because of the ability to synthesize extracellular matrix (ECM) tissue that play an important role in skin aging [15]. Evaluation of protein content after separation and purification confirmed that FTJ contains a significantly higher amount of protein than TJE and that the FTJ fraction contained more protein in the water layer than FTJ. Moreover, a pure compound was identified by preparative HPLC, and its structure and sequences were determined via NMR (1H, 13C, 2D-NMR). In the current study, we developed a new synthesis system which enabled the mass production of highly pure and stable bioactive materials. The new system was then used to synthesize a novel tripeptide (PEP) which displayed structural and sequential similarities to those of the peptides found in FTJ (Figure 1).

In this study, we investigated the effect of PEP on skin antiaging in human dermal fibroblasts (HDFs). Collagen primarily consists of extracellular matrix (ECM) proteins, which are responsible for the integrity, elasticity, and strength of human skin [26]. Degradation and reduction of collagen is closely linked to skin aging. Matrix metalloproteinases (MMPs), which are zinc-containing proteinases, play an important role in the process of skin aging via the degradation and modification of ECM proteins, which, in turn, are closely associated with inflammation, carcinogenesis, angiogenesis, and wound healing [27]. Among MMPs, MMP-1, a collagenase, degrades fibrillar collagen type I and III, while MMP-9, a gelatinase, breaks down ECM components such as collagen type I and IV in the skin. Reportedly, TNF-*α*, a major inflammatory cytokine, induces MMP-1 and MMP-9 expression and suppresses collagen synthesis in HDFs [28]. We confirmed that PEP was associated with cell proliferation and significant collagen synthesis, which also suppressed TNF-*α*-induced expression of MMP-1 and MMP-9 mRNA in HDFs (Figures 2–4).

We performed a cell migration assay to determine the effect of PEP on wound-healing activity in human keratinocytes. Migration and proliferation of keratinocytes at re-epithelialized wound sites are essential factors for tissue healing [29, 30]. We found that scratched HaCaT cells exhibited significant activity during wound healing when treated with 1 *μ*g/mL of PEP (Figure 5).

We performed a clinical study using an eye cream containing 0.5% PEP as a cosmetic ingredient. It was confirmed that application of PEP for 8 weeks improved wrinkle parameters involved in skin aging (Figure 6). A clinical trial was conducted using participants over the age of 40, who were concerned about wrinkles, sagging, laxity, and roughness of skin. The result suggested that PEP showed potential as a functional cosmetic product with antiaging effect. However, further studies may be needed to increase the sample size by increasing the number of volunteers to evaluate factors that play an important role in skin aging.

## 4. Conclusion

We synthesized a PEP with the same structure as that of the tripeptide derived from the fermented *T. japonica* fruit extract. We confirmed that the newly synthesized PEP exerted an effect on cell proliferation and collagen synthesis by decreasing the mRNA expression of TNF-*α*-induced MMP-1 and MMP-9 in HDFs. In addition, PEP promoted significant wound recovery in HaCaT cells and was found to enhance antiwrinkle effect clinically. These findings suggested that PEP showed potential as an antiskin-aging product which may be used in the cosmetic industry. However, this novel PEP may need further investigation, including clinical trials, in order to elucidate detailed molecular mechanisms underlying its effects and to enhance its efficacy as a skin product.

## Figures and Tables

**Table 1:** The components of the eye cream.

Component	Amount (% w/w)
EDTA-2Na	0.03
Glycerin	5.00
1,3-Butylene glycol	5.00
Sorbitol	5.00
Carbomer	0.30
Xanthan gum	0.30
Bees wax	1.50
Glyceryl stearate	0.50
Cetearyl alcohol	4.00
Phytosqualene	4.00
Carpylic/capric triglyceride	4.00
Shea butter	4.00
Dimethicone	2.50
Fragnance	0.40
1,2-Hexandiol	1.50
Tripeptide (5,000 ppm sol.)	0.5
Water q.s.	100

## Data Availability

No data were used to support this study.

## Abbreviations

TJE: *Trapa japonica* fruit extract.
